# Translating Evidence-Based Guidelines into Practice—Are We Getting It Right? A Multi-Centre Prospective International Audit of Nutrition Care in Patients with Foregut Tumors (INFORM)

**DOI:** 10.3390/nu12123808

**Published:** 2020-12-11

**Authors:** Merran Findlay, Judith D. Bauer, Rupinder Dhaliwal, Marian de van der Schueren, Alessandro Laviano, Adrianne Widaman, Lisa Martin, Andrew G. Day, Leah M. Gramlich

**Affiliations:** 1 Cancer Services, Royal Prince Alfred Hospital, Camperdown, NSW 2050, Australia; 2 Chris O’Brien Lifehouse, Camperdown, NSW 2050, Australia; 3 School of Human Movement and Nutrition Sciences, University of Queensland, St Lucia, QLD 4072, Australia; j.bauer1@uq.edu.au; 4 Canadian Nutrition Society, Ottawa, ON K1C 6A8, Canada; rupinder@cns-scn.ca; 5 Department of Nutrition, Dietetics and Lifestyle, School of Allied Health, HAN University of Applied Sciences, 6503 GL Nijmegen, The Netherlands; marian.devanderschueren@han.nl; 6 Department of Human Nutrition and Health, Wageningen University and Research, 6700 AA Wageningen, The Netherlands; 7 Department of Translational and Precision Medicine, Sapienza University of Rome, I-00185 Rome, Italy; alessandro.laviano@uniroma1.it; 8 Department of Nutrition, Food Science and Packaging, San Jose State University, San Jose, CA 95192, USA; adrianne.widaman@sjsu.edu; 9 University of California Davis Medical Center, Sacramento, CA 94558-5004, USA; 10 Department of Medicine, Cross Cancer Institute, University of Alberta, Edmonton, AB T6G 1Z2, Canada; ls2@ualberta.ca; 11 Department of Public Health Sciences, Queen’s University, Kingston, ON K7L 2V7, Canada; Andrew.Day@kingstonhsc.ca; 12 Royal Alexandra Hospital, Division of Gastroenterology, Department of Medicine, University of Alberta, Edmonton, AB T5H 3V9, Canada; lg3@ualberta.ca

**Keywords:** head and neck cancer, esophageal cancer, malnutrition, implementation, evidence-based practice, research translation

## Abstract

Malnutrition is highly prevalent in patients with foregut tumors comprising head and neck (HNC) and esophageal (EC) cancers, negatively impacting outcomes. International evidence-based guidelines (EBGs) for nutrition care exist; however, translation of research evidence into practice commonly presents considerable challenges and consequently lags. This study aimed to describe and evaluate current international nutrition care practices compared with the best-available evidence for patients with foregut tumors who are at high risk of malnutrition. A multi-centre prospective cohort study enrolled 170 patients commencing treatment of curative intent for HNC (*n* = 119) or EC (*n* = 51) in 11 cancer care settings in North America, Europe and Australia between 2016 and 2018. Adherence criteria were derived from relevant EBG recommendations with pooled results for participating centres reported according to the Nutrition Care Model at either system or patient levels. Adherence to EBG recommendations was: good (≥80%) for performing baseline nutrition screening and assessment, perioperative nutrition assessment and nutrition prescription for energy and protein targets; moderate (≥60 to 80%) for utilizing validated screening and assessment tools and pre-radiotherapy dietitian consultation; and poor (60%) for initiating post-operative nutrition support within 24 h and also dietetic consultation weekly during radiotherapy and fortnightly for 6 weeks post-radiotherapy. In conclusion, gaps in evidence-based cancer nutrition care remain; however, this may be improved by filling known evidence gaps through high-quality research with a concurrent evolution of EBGs to also encompass practical implementation guidance. These should aim to support multidisciplinary cancer clinicians to close evidence–practice gaps throughout the patient care trajectory with clearly defined roles and responsibilities that also address patient-reported concerns.

## 1. Introduction

Malnutrition is highly prevalent in patients with foregut tumors comprising head and neck (HNC) and esophageal (EC) cancers with rates commonly reported between 30 and 80% [1,2,3]. Characterized by a negative energy balance and skeletal muscle depletion, cancer-associated malnutrition is driven by a combination of reduced food intake and deranged metabolism [4]. Its etiology in patients with foregut tumors is complex, arising from factors related to: diagnosis (tumor location contributing to mechanical obstruction and tumor-induced metabolic derangements, cachexia and skeletal muscle catabolism); a prolonged, multi-modal treatment trajectory (surgery, radiotherapy and/or systemic therapy-related extensive nutrition-impact symptoms and treatment toxicities); and patient-related factors (age, pre-morbid nutritional status, socioeconomic circumstances and lifestyle factors such as alcohol and tobacco use) [5,6,7]. Patients can present as well-nourished at diagnosis, yet, due to the considerable disease and treatment burden, still develop the detrimental sequelae of malnutrition irrespective of overall weight status.

The best-available evidence to guide cancer nutrition care in practice has been synthesized in international evidence-based guidelines (EBGs) from Australia [8,9], Europe [10,11,12,13,14], North America [15] and the United Kingdom [16] (Table 1), demonstrating that optimizing nutrition care delivery leads to a positive impact on clinical, cost and patient-centred outcomes. These EBGs encompass nutrition care recommendations for varying aspects of the patient care trajectory and offer a focused synthesis by tumor site such as HNC [8,10,16] treatment modalities including radiotherapy and chemotherapy [9] and surgery [10,11,12,13] or cancer nutrition care [14,16] more broadly. A range of literature synthesis and quality appraisal methods [17,18,19,20,21] were employed in the development of the various EBGs; however, key recommendations are consistent. Despite the existence of EBGs, uptake of guidelines and translation of research evidence into practice lags up to 17 years in healthcare settings [22,23,24,25,26].

**Figure 1:**
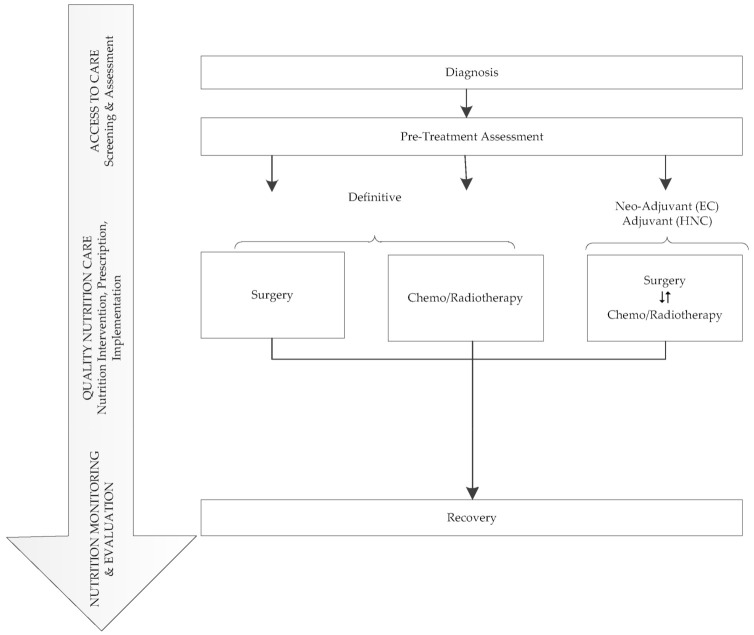
Overview of oncological treatment for patients with foregut tumors aligned with the Nutrition Care Model [27,28].

Underpinning quality nutrition care is the widely recognised Nutrition Care Model [27,28], comprising three domains: (1) Appropriate Access to Care (Nutrition Screening and Nutrition Assessment); (2) Quality Nutrition Care (Goals, Prescription, Implementation); and (3) Nutrition Evaluation and Monitoring (Measure and Evaluate Outcomes). Figure 1 depicts typical oncological treatment trajectories for patients with foregut tumors aligned with the Nutrition Care Model. In the first domain, elements of evidence-based nutrition care processes encompass use of validated tools for malnutrition screening and nutrition assessment such as the Subjective Global Assessment (SGA) [29] or Patient-Generated Subjective Global Assessment (PG-SGA) [30] specifically designed for use in oncology populations. Like the PG-SGA, the PG-SGA Short Form (PG-SGA SF) utilizes a patient-centric approach through self-identification of issues that impact nutrition yet still covers the conceptual domains of malnutrition and can therefore be used as a valid and reliable screening and monitoring instrument [31]. Patients with highly complex care needs require early and frequent access to dietitians with expertise in nutrition support; however, dietetic workforce resources in the cancer care setting are often inadequate [32,33,34].The second domain Quality Nutrition Care focuses on nutrition care processes around the timing and frequency of dietetic interventions, nutrition prescription, e.g., energy and protein and implementation such as commencing nutrition support, e.g., tube feeding. In the third domain, Measure and Evaluate Outcomes, EBGs seek to make recommendations regarding timing and frequency of dietetic interventions throughout the treatment trajectory and key nutrition elements and outcomes that should be monitored, e.g., weight, intake and nutritional status.

Successful implementation of research evidence into clinical practice requires identification of barriers to change and facilitators to overcome them in order for potential benefits of EBGs to be realized [35]. A Canadian-led international qualitative study exploring the barriers and enablers to nutrition care of patients with foregut tumors identified, firstly, a need to improve the evidence base and, secondly, to establish a minimum data set with a view to developing standardized nutrition care pathways with defined roles and responsibilities [36]. Recent Australian-led studies demonstrated that the multi-strategic implementation of innovative models of care increased adherence to EBGs for nutritional management of patients with HNC [8] resulted in improvements in nutritional status, quality of life [37], treatment completion, unplanned hospital admissions and associated costs of care [38]. This study aimed to (i) describe and evaluate current international nutrition care practices compared with the best-available evidence from published EBGs for patients with foregut tumors at high risk of malnutrition and (ii) determine any critical points where evidence–practice gaps persist and identify opportunities for improvement in nutrition care processes.

## 2. Materials and Methods

### 2.1. Study Design, Setting and Population

The International audit of Nutrition care in patients with FORegut tuMors (INFORM) multi-centre prospective cohort study was undertaken at 11 cancer care settings in Canada (*n* = 6 from two cities), Australia (*n* = 2), Italy (*n* = 1), the Netherlands (*n* = 1) and the United States of America (USA), (*n* = 1) between 2016 and 2018. Participating sites were teaching hospitals required to have a registered dietitian, clinical nutritionist or nutrition delegate available for study co-ordination. Inclusion criteria were adult (≥18 years) patients with a diagnosis of HNC or EC commencing treatment (any modality) of curative intent. Exclusion criteria were either absence of a treatment plan due to imminent death or an Eastern Co-operative Oncology Group (ECOG) [39] score ≥ 4.

### 2.2. Outcomes

The primary outcome was adherence to EBG nutrition care recommendations throughout the treatment trajectory for which adherence criteria based on international EBGs are presented in Table 2. Data regarding Appropriate Access to Care (Nutrition Screening and Assessment) are reported per usual practice at each site at the time of study enrollment and are therefore referred to here as system level outcomes within participating centres. The remainder of the adherence criteria are reported as a proportion of included patients and by participating site. The optimum degree of adherence to EBGs has not been determined; however, adherence rates of 80% to set criteria has been proposed [14] and applied in recent studies [37,38]. In this study, adherence to EBG recommendations was categorized as good (≥80%), moderate (≥60 to 80%) and poor (60%).

At study enrollment, each site provided cancer care setting demographics and details regarding which nutrition care processes were already established as part of standard care. Nutrition screening was defined as the process to identify an individual who is either malnourished or at risk of malnutrition to determine if a detailed nutrition assessment is indicated. Each study site reported their baseline practices regarding the validated tool or criteria used to identify patients who were malnourished or at risk of malnutrition. The validated tools for nutrition screening used in participating sites included the Malnutrition Screening Tool (MST) [40], Nutrition Risk Screening 2002 [41], Mini Nutrition Assessment-Short Form (MNA-SF) [42] and the Short Nutritional Assessment Questionnaire (SNAQ) [43]. Due to the variability in the types of tools that were used, the study protocol required participating sites to adopt the PG-SGA SF as the screening tool to facilitate longitudinal comparisons in nutrition risk across sites. To avoid misinterpretation of adherence data related to screening processes initiated as part of the study protocol (PG-SGA SF), each study site’s reported baseline practices were considered to be reflective of nutrition care processes with regards to screening and assessment. Nutrition assessment was defined as a comprehensive approach to diagnosing nutrition problems that use a combination of medical, nutrition, medication histories, physical examination, anthropometric measurements and laboratory data. Validated tools for comprehensive nutritional assessment for the purpose of evaluating nutritional status and, in particular, diagnosing the degree of malnutrition, if present, comprised both the PG-SGA [30] and SGA [29]. In this study, the criteria for comprehensive assessment of nutritional status was not considered to have been met where measures used in isolation, e.g., anthropometry alone were not assimilated as part of a comprehensive evaluation of nutritional status as outlined above.

Nutrition diagnosis is described as a critical step in the nutrition care model between assessment and intervention involving the identification and labeling of the specific nutrition problems that dietetics professionals are responsible for treating [44]. Examples of nutrition diagnoses include malnutrition (undernutrition), unintended weight loss, increased nutrient needs, inadequate protein-energy intake although there are many potential diagnoses that could be applied to the patient population studied here. Secondary outcomes were the impact of adherence to EBG recommendations on treatment completion and unplanned hospital admissions.

### 2.3. Data Collection

Research Electronic Data Capture (REDCap) [45] hosted at the University of Alberta, Canada, was used to capture data collected at each participating site from electronic and paper-based electronic medical records.

### 2.4. Statistical Analysis

All results are presented using descriptive statistics. Categorical variables are described as counts and percentages while continuous variables are described as medians and quartiles. All analysis was completed using SAS Version 9.4 (SAS Institute Inc., Cary, NC, USA).

### 2.5. Ethics Approval and Reporting

For Canadian locations as the international lead-site, ethics approval was obtained (HREBA.CC-15-0238). All other participating sites obtained ethics approval from their local ethics committee with Site-Specific Approval obtained for each of the participating sites, and patient consent was obtained. The study is reported according to the Strengthening the Reporting of Observational Studies in the Epidemiology (STROBE) checklist [46].

## 3. Results

### 3.1. Baseline Characteristics

Baseline patient, diagnosis and treatment characteristics are presented in Table 3. A total of 170 patients, with a diagnosis of either HNC (70.0%) or EC (30.0%) were recruited from 11 international sites. Patients were predominantly male (78.8%), Caucasian (94.7%) with a median [Q1, Q3] age of 62 [58, 69] years and an ECOG performance status ≤ 1 (92.8%). Tumor type was most commonly adenocarcinoma (80.4%) for patients with oesophageal cancer and the three most common tumor sites for HNC were oropharynx (32.8%), oral cavity (26.9%) and larynx (18.5%). Patients with HNC were more likely to have advanced clinical stage (III/IV) than patients with EC (77.4% versus 37.3%). Most patients (74.2%) received combined modality treatment in either adjuvant, neoadjuvant or definitive settings.

Baseline nutritional characteristics (Table 4) were similar for patients with either HNC or EC for both median (range) body mass index (BMI) kg/m^2^ (26 (14–42) versus 27 (17–39)) and nutritional risk as determined by median (range) PG-SGA SF score (7 (0–29) versus 7 (0–28)). A nutrition diagnosis was made for 50% of patients, with missing data (33.5%) likely reflective that formalizing this is not a universal practice amongst dietitians globally. The proportion of patients requiring nutrition support in terms of tube feeding during treatment was high for patients receiving surgery (70.3%) and radiotherapy (62.7%).

### 3.2. Adherence to Evidence-Based Guideline Recommendations

Adherence to EBG recommendations is presented in Table 5. For the first domain comprising Access to Care (Screening and Assessment), baseline nutrition care practices are as reported by each participating site. While malnutrition screening and nutrition assessment were reported to be routinely performed, validated tools were implemented for nutrition screening in 7/11 (64%) and for nutrition assessment in 8/11 (73%) of centres.

For the second and third domains of Quality Nutrition Care and Nutrition Monitoring and Evaluation, almost all patients undergoing treatment received nutrition assessment at some point during their care trajectory within the study period for either HNC (116/119, 98%) or EC (50/51, 98%). For patients undergoing surgery, nutrition assessment for patients with HNC was less frequent in the pre-operative setting 6/26 (23%) and more common in the post-operative setting 24/26 (92%) whereas, for patients with EC, nutrition assessment was high in both pre- and post-operative settings (37/38, 97% and 36/38, 95%). For patients undergoing either adjuvant or definitive radiotherapy or chemoradiotherapy, pre-treatment nutrition assessment occurred for 54/85 (64%) of patients with HNC and 27/41 (66%) of patients with EC. The proportion of patients receiving dietitian consultation each week during radiotherapy (RT) ranged from 69% to 88% for patients with HNC and 41% to 74% for patients with EC. The proportion of patients that received all recommended weekly dietitian assessments prescribed by evidence-based guidelines was 36/85 (42%) and 10/41 (24%) for patients with HNC and EC respectively. Nutrition prescription for energy (kcal/kg) and protein (g/kg) was similar between the surgical and radiotherapy settings in both the HNC and EC groups (Table 5).

### 3.3. Unplanned Admissions, Treatment Completion and Survival

Rates of unplanned admission, treatment completion and survival were similar between EC and HNC groups. The unplanned admission rate was slightly higher for patients with EC than HNC (21/51 (41%) versus 33/119 (28%)). The overall treatment completion rate was 139/170 (82%) and was similar between EC and HNC groups (43/51 (84%) versus 96/119 (81%). At the end of the study, the overall proportion of patients surviving was 157/170 (92%) with no difference between EC and HNC groups (45/51 (88%) versus 112/119 (94%).

## 4. Discussion

This is the first study to prospectively explore adherence to evidence-based nutrition care processes in an international multi-centre cohort of high-nutritional-risk patients with HNC and EC. By the adherence benchmarks described in our methods, the participating sites adhered well (80%) to recommendations for completing nutrition screening and assessment, perioperative nutrition assessment and nutrition prescription for energy and protein targets. Adherence was moderate (≥60 to 80%) for utilizing validated screening and assessment tools and pre-radiotherapy dietitian consultation and poor (60%) for initiating post-operative nutrition support within 24 h and also dietetic consultation occurring weekly during radiotherapy and fortnightly for 6 weeks post-radiotherapy.

With regards to Quality Nutrition Care and Nutrition Monitoring and Evaluation, at a patient population or system level, the proportion of patients that received dietetic assessment from week to week ranged from 69% to 88% for patients with HNC and 41% to 74% for patients with EC. However, patient-level data revealed the evidence-based schedule of weekly dietitian consultations was adhered to for 42% of patients with HNC and 24% of patients with EC. This tapered further for the recommended fortnightly for six weeks post-treatment dietitian consultations to 13% and 17%, respectively. Our patient-level data concur with the EBGs suggesting that weight loss and nutrition impact symptoms highlight the importance of ongoing access to nutrition care. Recommendations regarding perioperative nutrition assessment were largely well adhered to; however, nutrition intervention in terms of initiating post-operative nutrition support within 24 h was sub-optimal. Reasons for low adherence with some guideline recommendations were not captured within the scope of this study; however, given the tendency for variation in individual and institutional practices, exploration of local barriers and facilitators to improving EBG adherence within participating centres may be warranted.

The benefits of successful implementation of evidence-based nutrition care in line with best-practice in high-risk tumor groups are now well-documented. Australian-led implementation studies have demonstrated adherence to an evidence-based schedule of dietitian visits throughout treatment for HNC and recovery leads to improved outcomes for patients including nutritional status, weight maintenance, quality of life, treatment completion, and unplanned admissions [37,38,47]. Britton et al. [37,48] and McCarter et al. [47,49] uniquely demonstrated that dietitian-led delivery of motivational interviewing and cognitive behavioral therapy as part of an Eating as Treatment (EAT) intervention improved a range of patient outcomes. From a system-level perspective, Findlay et al. [38] demonstrated a 15% relative reduction in costly unplanned hospital admissions equating to (in Australian Dollars (AUD)) 14.65 AUD in costs avoided for every 1 AUD invested in delivering an evidence-based model of care to patients with HNC. Further, patients who required unplanned hospital admission due to their deteriorating condition utilized approximately twice the dietetics resources over the course of care with a median (range) number of dietitian consultations of 15 (3–66) versus 8 (2–29), *p* 0.001. Inadequate dietetic resources are frequently cited as a key barrier to delivering optimum nutrition care [33]; however, alongside medical and nursing expertise, allied health professionals are the third pillar of the health workforce [50] and, with the required upfront organizational investment, have the ability to deliver high value care.

Overcoming local barriers to guideline implementation, however, can be challenging and requires a multi-strategic approach. Martin et al. [36] have highlighted the need for standardized nutrition care pathways with clearly defined roles and responsibilities. Further, the prognostic significance of cancer-related malnutrition and sarcopenia [51,52,53,54,55,56,57,58,59,60] in these patients warrants specific attention. The Clinical Oncology Society of Australia (COSA) have recently articulated a Position Statement to support the implementation of a co-ordinated, multidisciplinary pathway for screening, assessment and treatment of these conditions [61]. A culture of review and audit is a longstanding mainstay of quality in healthcare and the availability of EBGs to guide cancer nutrition care represent an opportunity to establish this amongst cancer nutrition professionals as integral to overall quality in cancer care.

Our prospective audit was based on adherence criteria derived from the best-available evidence; however, EBGs are not without limitations, particularly when evidence quality is low or evidence gaps persist. As with all scientifically rigorous processes, the appropriateness of some EBG recommendations should be challenged. An example highlighted in our audit is the optimum nutrition prescription in terms of protein and energy targets. Different guidelines recommend a daily energy prescription of either 125 kJ/kg (30 kcal/kg) [8,9] or 25–30 kcal/kg [12,14]; however, a recent study suggests that the lower end of this range (25 kcal/kg/day) specified in some EBGs may be insufficient to maintain positive energy balance and therefore ameliorate skeletal muscle depletion in patients with HNC [62]. Of note, the EFFORT trial (effect of early nutritional support on frailty, functional outcomes, and recovery of malnourished medical inpatients) implemented a systematic approach to individualized nutritional support in high-risk inpatients which demonstrated improved clinical outcomes, including survival [63]. However, it should also be acknowledged that merely increasing energy and protein targets is an overly simplistic view of what is frequently an enormously challenging aspect of self-managed care for ambulatory patients. Reasons for reduced nutritional intake are multifactorial and require multidisciplinary management to create a path to support patients to optimize nutrient intake. In an exploration of patient-reported barriers to following tube feeding prescription in patients with HNC despite intensive dietetic input, Brown et al. [64] concluded that understanding patient needs and optimizing multidisciplinary symptom management are vital to improving adherence to nutrition care recommendations. This prompts a call to action for high-quality research to fill known evidence gaps, and, as new findings to guide cancer nutrition care are published, EBGs and related recommendations subsequently require ongoing updates and should be accompanied by implementation guidance with clearly defined roles and responsibilities for multidisciplinary teams that also address patient-reported barriers.

Study strengths include the international perspective on adherence to evidence-based practice with a focus on tertiary referral centers that routinely manage high volumes of patients undergoing treatment for complex, high-nutritional risk cancers. In terms of limitations, the authors acknowledge the convenience sampling and lack of ethnic diversity in the study population may have resulted in selection bias. Despite the increasingly cultural and linguistic diversity of Western countries within which the study sites were located, the predominantly Caucasian study participants may be reflective of the challenges of recruiting cohorts that are representative of the broader population to research participation. Further, where data were reported at the system level, a truer reflection of sustained implementation of best-practice nutrition care processes may have been captured with whether adherence occurred at each nutrition intervention time point. Finally, the sample size was relatively small and the 11 participating sites may not be reflective of the practice at other sites.

## 5. Conclusions

From an international perspective, adherence to evidence-based guideline recommendations in line with best-practice nutrition care of patients with HNC and EC was: good for baseline nutrition screening and assessment processes, perioperative nutrition assessment and nutrition prescription for energy and protein; moderate for utilizing validated screening and assessment tools and pre-radiotherapy dietitian consultation; and poor for initiating post-operative nutrition support within 24 h, weekly dietetic consultation during radiotherapy and fortnightly for 6 weeks post-radiotherapy. This demonstrates there are still evidence to practice gaps in a global uptake of research evidence into practice for some aspects of care, highlighting areas for improvement. Where evidence–practice gaps persist, a systematic, multi-strategic approach that supports local implementation is required to better link research and practice in order to ultimately optimize patient care and outcomes.

## Figures and Tables

**Table 1:** Evidence-based clinical practice guidelines for nutritional management of adults with cancer.

First Author, Year	Origin	Organisation	Guideline Focus	Appraisal Method
Arends, 2017 [14]	Europe	ESPEN ^a^	Cancer	GRADE ^b^
Dort, 2017 [10]	International	ERAS ^c^ Society	Surgery–HNC ^d^	GRADE
Thompson, 2017 [15]	USA ^e^	AND ^f^	Cancer	AND EAL ^g^
Weimann, 2017 [11]	Europe	ESPEN	Surgery	SIGN ^h^, AHCPR ^i^
Talwar, 2016 [16]	UK ^j^	BAHNO ^k^	HNC	ns ^l^
Isenring, 2013 [9]	Australia	DAA ^m^	RT ^n^, CTx ^o^	NHMRC ^p^
Findlay, 2011 [8]	Australia *	COSA ^q^	HNC	NHMRC
Braga, 2009 [12] **	Europe	ESPEN	Surgery-PN ^r^	ns
Weimann, 2006 [13] **	Europe	ESPEN	Surgery	ESPEN

^a^ ESPEN = European Society for Clinical Nutrition and Metabolism; ^b^ GRADE = Grading of Recommendations Assessment Development and Evaluation; ^c^ ERAS = Enhanced Recovery After Surgery; ^d^ HNC = Head and Neck Cancer; ^e^ USA = United States of America; ^f^ AND = American Academy of Nutrition and Dietetics; ^g^ EAL = Evidence Analysis Library; ^h^ SIGN = Scottish Intercollegiate Guidelines Network; ^i^ AHCPR = Agency for Health Care Policy and Research; ^j^ UK = United Kingdom; ^k^ BAHNO = British Association of Head and Neck Oncologists; ^l^ ns = not specified; ^m^ DAA = Dietitians Association of Australia; ^n^ RT = Radiotherapy; ^o^ CTx = Chemotherapy; ^p^ NHMRC = National Health and Medical Research Council; ^q^ COSA = Clinical Oncology Society of Australia; ^r^ PN = Parenteral Nutrition; * Endorsed by Dietitians Association of Australia, Dietitians New Zealand, British Dietetics Association and available via Dietitians Canada Practice-Based Evidence in Nutrition online portal; ** Merged into Weimann et al., 2017 [11].

**Table 2:** Adherence criteria for evidence-based guideline recommendations.

Nutrition Care Model [28,29]	Recommendation	Guideline	Evidence *	Adherence Criteria
ACCESS TO CARE Screening Assessment	Malnutrition screening should be undertaken on all patients at diagnosis to identify nutritional risk and then repeated at intervals through each stage of treatment (eg surgery, radio/chemotherapy and post-treatment).	COSA ^a^ [8], DAA ^b^ [9] ESPEN ^c^, 2017 [14] AND ^d^ [15] ESPEN, 2017 [11]	B V Low/Strong Strong/Imperative Not specified	Malnutrition screening occurred as part of standard care at site prior to study enrollment.
	All patients receiving radiotherapy to the gastrointestinal tract or head and neck area and should be referred to the dietitian for nutrition support.	DAA, COSA	A	Dietetic consult occurred before week 1 of radiotherapy.
	Use a validated nutrition screening tool (e.g., MST ^e^) for identifying malnutrition risk.	DAA, COSA AND	B Strong/Imperative	Use of validated screening tool occurred as part of standard care at site prior to study enrollment.
	Use a validated nutrition assessment tool (e.g., PG-SGA ^f^ or SGA ^g^).	DAA, COSA AND	B Strong/Imperative	Use of validated nutrition assessment tool occurred when assessing nutritional status.
QUALITY NUTRITION CARE Nutrition intervention, prescription, implementation	*Intervention* Weekly dietitian contact improves outcomes in patients receiving radiotherapy.	DAA, COSA	A	Dietetic consult occurred at least once for every five fractions of radiotherapy given in a single working week period.
	*Prescription* RT ^h^: Aim for energy intakes of at least 30 kcal/kg/day (125 kJ/kg/day) and 1.2 g protein/kg/day in patients receiving radiotherapy.	DAA, COSA	C	*Radiotherapy* Estimated Energy Requirements (kcal or kJ/kg/day) Estimated Protein Requirements (g/kg/day)
	Surgery: Aim for Energy intakes of at least 30 kcal/kg/day (125 kJ/kg/day)/25–30 kcal/kg IBW ^i^/day. Protein intake of 1.5 g/kg IBW in illness/stress.	COSA ESPEN 2017 [14] ESPEN 2009 [12] ESPEN 2009 [12]	C Low/Strong B B	*Surgery* Estimated Energy Requirements (kcal or kJ/kg/day) Estimated Protein Requirements (g/kg/day)
	*Implementation* Surgery: Post-operative tube feeding should commence within 24 h.	COSA ERAS ^j^ [10] ESPEN 2017 [11]	A Mod/Strong A/GPP ^k^	Post-operative nutrition support commenced within 24 h.
NUTRITION MONITORING EVALUATION	Patients should be seen weekly by a dietitian during radiotherapy.	DAA, COSA	A	As above
	Patients should receive minimum fortnightly follow up by a dietitian for at least 6 weeks post radiotherapy.	DAA, COSA	A	Dietetic consult occurred at least once in a 14 day period following end of radiotherapy for three consecutive fortnights.

* Evidence rating according to appraisal method specified in individual evidence-based guideline. ^a^ COSA = Clinical Oncology Society of Australia; ^b^ DAA = Dietitians Association of Australia; ^c^ ESPEN = European Society of Clinical Nutrition and Metabolism; ^d^ AND = Academy of Nutrition and Dietetics; ^e^ MST = Malnutrition Screening Tool; ^f^ PG-SGA = Patient-Generated Subjective Global Assessment; ^g^ SGA = Subjective Global Assessment; ^h^ RT = Radiotherapy; ^i^ IBW = Ideal Body Weight; ^j^ ERAS = Enhanced Recovery After Surgery; ^k^ GPP = Good Practice Point.

**Table 3:** Baseline characteristics.

Characteristic	Head and Neck (*n* = 119) *n* (%)	Esophageal (*n* = 51) *n* (%)	All (*n* = 170) *n* (%)
Centre (Country–City)			
Australia–Brisbane	10 (8.4%)	0 (0.0%)	10 (5.9%)
Australia–Sydney	20 (16.8%)	0 (0.0%)	20 (11.8%)
Canada-Calgary *	21 (17.6%)	10 (19.6%)	31 (18.2%)
Canada–Edmonton *	20 (16.8%)	18 (35.3%)	38 (22.4%)
Italy–Rome	19 (16.0%)	0 (0.0%)	19 (11.2%)
Netherlands-Amsterdam	20 (16.8%)	23 (45.1%)	43 (25.3%)
USA-Sacramento	9 (7.6%)	0 (0.0%)	9 (5.3%)
Age, years			
median [Q1, Q3]	62 [58, 69]	65 [56, 71]	63 [57, 70]
Sex			
Male	93 (78.2%)	41 (80.4%)	134 (78.8%)
Female	26 (21.8%)	10 (19.6%)	36 (21.2%)
Ethnicity			
Caucasian	111 (93.3%)	50 (98.0%)	161 (94.7%)
First Nations	1 (0.8%)	0 (0.0%)	1 (0.6%)
Hispanic	2 (1.7%)	0 (0.0%)	2 (1.2%)
Asian	3 (2.5%)	1 (2.0%)	4 (2.4%)
East Indian	1 (0.8%)	0 (0.0%)	1 (0.6%)
Other	1 (0.8%)	0 (0.0%)	1 (0.6%)
Current Smoker			
Yes	44 (37.0%)	13 (25.5%)	57 (33.5%)
No	75 (63.0%)	38 (74.5%)	113 (66.5%)
Alcohol use			
Yes	47 (39.5%)	9 (17.6%)	56 (32.9%)
No	72 (60.5%)	42 (82.4%)	114 (67.1%)
ECOG ^a^ Performance Status			
0	78 (65.5%)	32 (62.7%)	110 (64.7%)
1	32 (26.9%)	16 (31.4%)	48 (28.2%)
2	6 (5.0%)	3 (5.9%)	9 (5.3%)
3	3 (2.5%)	0 (0.0%)	3 (1.8%)
Clinical Stage			
1	5 (4.2%)	4 (7.8%)	9 (5.3%)
2	8 (6.7%)	13 (25.5%)	21 (12.4%)
3	18 (15.1%)	15 (29.4%)	33 (19.4%)
4 (Any)	74 (62.2%)	4 (7.8%)	78 (45.9%)
Could not assess stage	7 (5.9%)	11 (21.6%)	16 (10.6%)
Not staged	7 (5.9%)	4 (7.8%)	11 (6.5%)
Tumor Site–Head and Neck			
Primary unknown	3 (2.5%)		3 (1.8%)
Hypopharynx	10 (8.4%)		10 (5.9%)
Larynx	22 (18.5%)		22 (12.9%)
Nasopharynx	5 (4.2%)		5 (2.9%)
Oral cavity	32 (26.9%)		32 (18.8%)
Oropharynx	39 (32.8%)		39 (22.9%)
Other	3(2.5%)		3 (1.8%)
Salivary gland	5 (4.2%)		5 (2.9%)
Treatment Modality ^b^			
None	5 (5.0%)	5 (9.8%)	10 (6.6%)
Chemotherapy-definitive	0 (0.0%)	2 (3.9%)	2 (1.3%)
Chemotherapy-adjuvant	0 (0.0%)	1 (2.0%)	1(0.7%)
Radiotherapy-definitive	15 (15.0%)	0 (0.0%)	15 (9.9%)
Surgery	10 (10.0%)	2 (3.9%)	12 (7.9%)
Chemoradiotherapy-definitive	54 (54.0%)	6 (11.8%)	60 (39.7%)
Surgery + adj ^c^/neoadj ^d^ RT ^e^	7 (7.0%)	0 (0.0%)	7 (4.6%)
Surgery + adj/neoadj CRT ^f^	9 (9.0%)	35 (68.6%)	44 (29.1%)

* 3 participating sites each from Edmonton and Calgary. ^a^ ECOG = European Co-Operative Group; ^b^ Treatment modality–19 patients from one site (Rome, Italy) excluded because cancer treatment was not captured; ^c^ adj = adjuvant; ^d^ neoadj = neoadjuvant; ^e^ RT = Radiotherapy; ^f^ CRT = Chemoradiotherapy.

**Table 4:** Baseline nutritional characteristics.

Nutritional Characteristics		Head and Neck (*n* = 119) *n* (%)	Esophageal (*n* = 51) *n* (%)	All (*n* = 170) *n* (%)
Weight (kg)				
	median [Q1, Q3]	77 [67, 91]	85 [75, 95]	81 [70, 91]
Height (cm)				
	median [Q1, Q3]	173 [168, 178]	176 [170, 182]	174 [169, 179]
BMI ^a^ (kg/m^2^)				
	median [Q1, Q3]	26 [23, 30]	27 [25, 31]	26 [23, 30]
Was a nutrition diagnosis made?				
	Yes	52 (43.7%)	33 (64.7%)	85 (50.0%)
	No	27 (22.7%)	1 (2.0%)	28 (16.5%)
	No data	40 (33.6%)	17 (33.3%)	57 (33.5%)
Nutritional Risk, PG-SGA ^b^ Short Form Score				
	(n) median [Q1, Q3]	(116) 5 [1, 10]	(47) 8 [4, 11]	6 [2, 11]
Location patient first introduced to cancer care setting				
	Inpatient	14 (11.8%)	4 (7.8%)	18 (10.6%)
	Outpatient	105 (88.2%)	47 (92.2%)	152 (89.4%)
Did patient receive EN ^c^ or PN ^d^ within the last month prior to study enrollment?				
	Yes	16 (13.4%)	6 (11.8%)	22 (12.9%)
	No	103 (86.6%)	45 (88.2%)	148 (87.1%)

^a^ BMI = Body Mass Index; ^b^ PG-SGA = Patient-Generated Subjective Global Assessment; ^c^ EN = Enteral Nutrition; ^d^ PN = Parenteral Nutrition.

**Table 5:** Adherence to evidence-based guideline recommendations. Access to care (screening and assessment) by the total number of participating sites (*n* = 11); quality nutrition care and nutrition monitoring and evaluation by the total number of patients (*n* = 170).

Nutrition Care Model [27,28]	Measure–System Level (Usual Practice in Centre at Study Enrollment)	N (*n* = 11)		%	
Access to Care	**Screening**				
	Malnutrition screening routinely performed.	11		100%	
	Validated nutrition screening tool used.	7		64%	
	**Assessment**				
	Nutrition assessment routinely performed.	11		100%	
	Validated nutrition assessment tool used.	8		73%	
Quality Nutrition Care	**Measure—Patient Level**	**HNC**		**EC**	
	**Surgery**	**(*n* = 26)**		**(*n* = 38)**	
		***n***	**%**	***n***	**%**
	Nutrition assessment				
	Pre-operative	6	23%	37	97%
	Post-operative	24	92%	36	95%
	Nutrition support-tube feeding commenced				
	Pre-operative	1	4%	8	21%
	Post-operative				
	within 24 h	5	19%	2	5%
	within 1 to 7 days	12	46%	17	44%
	Nutrition prescription, median [Q1, Q3]				
	Energy prescription (kcal/kg/d)	30.8 [29.6, 32.7]		26.3 [23.2, 28.1]	
	Protein prescription (g/kg/d)	1.3 [1.2, 1.5]		1.4 [1.2, 1.5]	
	**Radiotherapy/Chemoradiotherapy**	**(*n* = 85)**		**(*n* = 41)**	
		***n*^#^**	**%**	***n*^#^**	**%**
	Nutrition prescription, median [Q1, Q3]				
	Energy prescription (kcal/kg/d)	30.4 [26.7, 33.4]		26.7 [23.3, 28.8]	
	Protein prescription (g/kg/d)	1.3 [1.2, 1.5]		1.4 [1.2, 1.5]	
Nutrition Monitoring and Evaluation	Received recommended dietitian consultation:				
	Pre-treatment (Access to Care)	54/85	64%	27/41	66%
	Weekly during treatment	36/85	42%	10/41	24%
	Fortnightly for 6 weeks post-treatment	11/85	13%	17/41	17%

# denotes diminishing n-value over course of RT duration.
